# Meningiomas of the Cerebellopontine Angle: Radiological Differences in Tumors with Internal Auditory Canal Involvement and Their Influence on Surgical Outcome

**DOI:** 10.1371/journal.pone.0122949

**Published:** 2015-04-07

**Authors:** Kun Gao, Housheng Ma, Yong Cui, Xuzhu Chen, Jun Ma, Jianping Dai

**Affiliations:** 1 Department of Interventional Radiology, Beijing Tiantan Hospital, Capital Medical University, Beijing, People’s Republic of China; 2 Department of Radiology, Yantai Yuhuangding Hospital, Yantai, Shandong Province, People’s Republic of China; 3 Department of Neurosurgery, Beijing Tiantan Hospital, Capital Medical University, Beijing, People’s Republic of China; 4 Department of Neuroradiology, Beijing Tiantan Hospital, Capital Medical University, Beijing, People’s Republic of China; University of Manchester, UNITED KINGDOM

## Abstract

This study explored the clinical, radiological, and pathological characteristics of cerebellopontine angle (CPA) meningiomas with internal auditory canal (IAC) involvement. The pre- and postoperative MR images of 193 consecutive patients with pathologically diagnosed meningioma centered around the IAC were analyzed, focusing on changes in the IAC, maximal axial tumor volume, peritumoral brain edema, and postoperative residual tumor. Patient age, sex, tumor volume, postoperative residual tumor, and pathological subtype were compared in patients with and without IAC involvement by the tumor and among the different types of IAC involvement. The results showed that the 71 patients (36.8%) with IAC involvement had a higher ratio of peritumoral edema (χ^2^=5.922, *P*=0.015), postoperative residual tumor (χ^2^=22.183, P< 0.001), and a predominance of the meningothelial subtype (χ^2^=5.89, *P*=0 .015). Peritumoral edema was a risk factor for IAC involvement (*P*=0.016, OR=2.186). Radiologically, IAC involvement could be distinguished as intruding (31%, 22/71), filled (29.6%, 21/71), and dilated (39.4%, 28/71). Patients with intruding IAC were significantly older (54.5±9.54 years, *P*=0.021) and had the lowest postoperative residual tumor values (42%, χ^2^=7.865, *P*= 0.005), while those with filled IAC were more likely to be female (95%, χ^2^=9.404, *P*=0.009).Our observations provide the basis for a morphological classification of IAC involvement by CPA meningiomas and further insight into the clinical features of these tumors.

## Introduction

Meningiomas account for 26% of primary intracranial neoplasms[1]. Among intracranial meningiomas, 5%–10% are located in the cerebellopontine angle (CPA) [2, 3], thus comprising the second most common tumors in this anatomic region [3]. Although enlargement and involvement of the internal auditory canal (IAC) is a common sign of schwannomas of the CPA [2, 4], these features can also be seen in meningioma[5]. Meningiomal involvement of the IAC occurs in two forms: meningioma truly originating in the IAC and meningioma extending into the IAC from an adjacent location. While the first form is very rare, the second is relatively common [6–10].

The location of a CPA meningioma affects clinical outcome [11]. Patients with CPA meningiomas with IAC involvement often present with hearing loss and abnormal facial motor function. Resection of these tumors is compatible with hearing restoration [12]. Nonetheless, the anatomical location and intimate relationship between meningiomas involving the IAC and the cranial nerves are such that skilled surgical management is needed. Despite advances in neurosurgery, the surgical procedure for CPA meningiomas involving the IAC remains challenging [11, 13].

Studies on CPA meningiomas with IAC involvement have focused, for example, on functional preservation during surgery [8], the effects of radiotherapy [10], angiographic findings [13], and skull-base bone involvement [4, 14]. However, the extent of IAC involvement of these tumors, their morphological types, and their influence on tumor surgical resection have hardly been evaluated. Therefore, in this study, we investigated the different radiological subtypes of invasive meningioma involving the IAC and the amount of postoperative residual tumor associated with each one in a cohort of 193 patients with CPA meningioma.

## Materials and Methods

This retrospective study was reviewed by the Review Board of Beijing Tiantan Hospital, Capital Medical University. The informed consent requirement was waived as the patients’ records/information were anonymized and de-identified prior to the analysis.

### Patients

**Fig 1:**
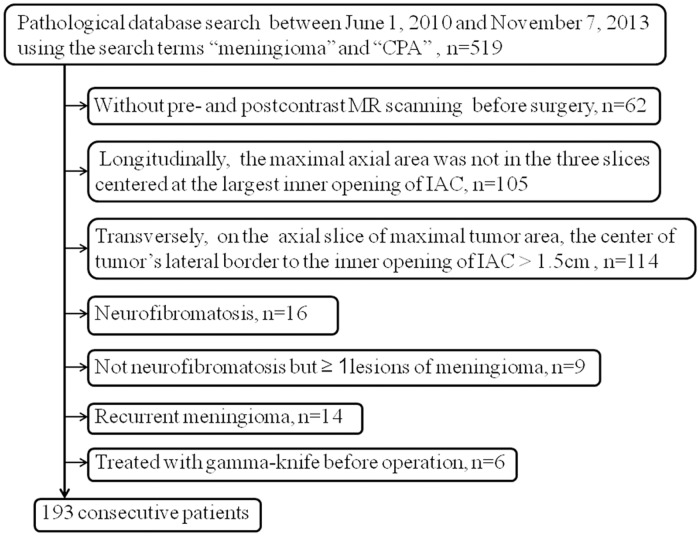
Flow chart of the patient cohort.

A computerized search of the pathological database of our institute between June 1, 2010 and November 7, 2013 was performed using the search terms “meningioma” and “CPA”, which yielded 519 patients. The inclusion criteria were as follows: 1) pathological diagnosis of meningioma after surgery; 2) MRI of the head before surgery; 3) detection on preoperative MRI of the maximal transverse area of the tumor, either on the axial slice showing the widest inner opening of IAC or on a closely adjacent slice; and 4) on the axial slice showing the maximal tumor area, a distance of ≤1.5cm between the center of tumor’s lateral border and the inner opening of the IAC. The exclusion criteria are shown in Fig 1. After the exclusion of 326 patients, 193 patients (mean age, 50.2yeras; range, 19–72 years) comprising 39 men (mean age, 50.4 years; range, 29–72 years) and 154 women (mean age, 50.1 years; range, 19–71 years) were included in this study. The case accrual process is summarized in Fig 1.

### MRI parameters

All preoperative MRI examinations were performed within seven days of surgery. The protocol included unenhanced and enhanced sequences. The pre-contrast sequence consisted of axial T1WI, T2WI, and sagittal T1WI. Once the pre-contrast imaging was completed, 0.2 mL of gadopentetate dimeglumine (Magnevist; BayerHealthCare Pharmaceuticals, Wayne, NJ, USA)/kg was injected manually into the patient’s antecubital vein by a registered nurse. Post-contrast images, including the axial, sagittal, and coronal images, were obtained immediately after the administration of contrast media. A Genesis Signa 3T scanner (GE Healthcare, Milwaukee, WI, USA) was used to scan 88 patients. T1WI (TR/TE, 2031ms/19ms) and fast spin echo (FSE) T2WI (TR/TE, 4900ms/116–123ms) sequences were obtained with a field of view (FOV) of 240mm and a 512×512 matrix. Another 3T superconducting MR scanner (Magnetom, Trio; Siemens, Erlangen, Germany) was used to image 45 patients. T1WI turbo inversion recovery sequence (TR/TE, 2000ms/9.8ms) with a 512×432 matrix and T2WI turbo spin-echo sequence (TR/TE, 4500ms/84ms) with a 384×324 matrix were obtained. In 24 patients, a 3T Siemens Verio scanner was used to acquire T1WI dark fluid sequence (TR/TE, 2400ms/9.4ms) with a 512×496 matrix and T2WI sequence (TR/TE, 6000ms/97ms) with a 640×640 matrix. The remaining 36 patients were scanned on a 1.5T Signa HDe MR scanner (GE Healthcare). T1WI (TR/TE, 2031.25ms/19.5ms) and T2WI (TR/TE, 4900ms/123.62ms) sequences with the same FOV (240mm) and matrix (512×512) were obtained. For all 193 patients, the scanning section thickness and gap were 5mm and 6mm, respectively. Postoperative MRI was done within seven days postoperatively using the same scanning protocol.

### MRI analysis and measurement

Peritumoral brain edema was defined as patchy hypointensity on T1WI images and hyperintensity on T2WI images in the ipsilateral cerebellar hemisphere, vermis and/or brain stem, surrounding the medial margin of the tumor.

Three types of IAC involvement were defined. In the intruding form, part of the tumor extended into the IAC such that it occupied a small part of the canal, with no enlargement of the inner opening of IAC. In the filled form, all or a major part of the IAC was replaced by the tumor but without expansion of the IAC. In the dilated form, the inner opening of the tumor-occupied IAC was larger than on the contralateral normal side. A bilateral comparison of the IACs was based on bilateral measurements (in mm) of the inner opening on axial T2WI slices showing the largest IACs. The tumor volume was determined based on the maximal two-dimensional area (mm^2^) on axial post-contrast images. Measurement of the inner opening of the IAC and axial tumor volume were semi-automatically performed using the software Neusoft PACS/RIS version 3.1 (http://pacs.neusoft.com). The presence of residual tumor was recorded if, on postoperative MRI, the ipsilateral IAC was partly or fully filled by abnormal signal intensity (with the same signal intensity as on preoperative pre- and post-contrast MRI).

IAC involvement, infratentorial peritumoral brain edema, and postoperative residual tumor were independently confirmed by two radiologists (one with 22 years and the other with 18 years of experience) who also measured the inner opening of the IAC and the area of the tumor. Both radiologists were blinded to each other’s findings and to the pathologic information. Discrepancies regarding IAC involvement, peritumoral edema, and postoperative residual tumor were resolved by consensus. The average tumor area was determined by the two observers and was considered as the final tumor volume in the statistical analysis.

### Pathological diagnosis

The surgical specimen was processed to confirm a diagnosis of meningioma and then subjected to immunohistochemistry to identify the pathological subtypes and malignant grades, according to the 2007 World Health Organization (WHO) criteria [15].

### Statistical analysis

The image data from the 193 patients was divided into four subsets according to the different preoperative MR scanner used. Comparison among the four subsets were carried out using a one-way analysis of variance (ANOVA) for patient age and the χ^2^ for patient sex and tumor volume. Based on IAC involvement, the patients were then further divided into two groups: those with IAC involvement (n = 71) and those without (n = 122). Patient age was compared using the independent- sample *t* test; the sex of patient, peritumoral edema, postoperative residual tumor, and pathological subtype of the tumor were compared using the χ^2^ test. Because tumor volume data did not follow a normal distribution, they were analyzed using a nonparametric two-independent-samples test. The 71 patients with IAC involvement were then divided into three groups, according to the type of IAC invasion. Among the three groups, patient age was compared using ANOVA; patient sex, peritumoral edema, postoperative residual tumor, and pathological subtypes were compared using the χ^2^ test; and tumor volume using a nonparametric K independent samples test. Binary logistic regression was then used to test whether patient age, sex, tumor volume, peritumoral edema, or pathological subtype were related to IAC involvement. Statistical analysis was done using commercial statistical software (SPSS 13.0, SPSS Inc, Chicago, IL, USA). A *P* value less than 0.05 was considered statistically significant.

## Results

Among the 193 cases of meningiomas, 31.6% (61/193) were meningothelial, 22.8% (44/193) were fibrous, 22.3% (43/193) were mixed, 8.8%(17/193) were transitional, 5.2%(10/193) were angiomatous, 4.1%(8/193) were secretory, 1.6%(3/193) were chordoid, 1%(2/193) were clear cell, and another 1%(2/193) were atypical. In three tumors (1.6%), no definite subtype could be determined. Based on the WHO classification [15], the majority (94.8%, 183/193) of the tumors were grade I whereas only 10 (5.2%) tumors were grades II and III.

In a comparison of the four subsets of patients with respect to the MR scanner, there was no significant difference in patient age (*P* = 0.684) or sex (χ^2^ = 0.814, *P* = 0.846) or tumor volume (χ^2^ = 0.473, *P* = 0.925), indicating that the data were not biased because of the different MR scanners. There were also no differences between patients with (37%, 71/193) and without IAC involvement by CPA meningioma with respect to tumor volume (*P* = 0.898), patient age (*P* = 0.452), and sex (χ^2^ = 1.844, *P* = 0.174). However, patients with IAC involvement had more extensive peritumoral edema (χ^2^ = 5.922, *P* = 0.015) and postoperative residual tumor (χ^2^ = 22.183, *P*< 0.001) and a predominance of the meningothelial subtype (χ^2^ = 5.89, *P* = 0.015) (Table 1).

**Table 1:** Characteristics of the patients with and without IAC involvement by meningioma of the cerebellopontine angle.

	Patients with IAC involvement (n = 71)	Patients without IAC involvement (n = 122)	*P* value	χ2 value
**Age**: in years (mean, range)	49.44±10.45, 29–72	50.58±10.28, 19–69	*P* = 0.452	
**Female patients**: n (%)	53/71 (75)	101/122 (83)	*P* = 0.174	χ^2^ = 1.844
**Axial tumor volume in mm** ^**2**^ median (25–75%)	629 (406–932.75)	594.5 (413–954)	*P* = 0.898*	
**Peritumoral edema**: n (%)	28/71 (39)	28/122 (23)	*P* = 0.015	χ^2^ = 5.922
**Postoperative residual tumor**: n (%)	37/52 (71)	25/84 (30)	*P*<0.001**	χ^2^ = 22.183
**Meningothelial subtype**: n (%)	30/71 (42)	31/122 (25)	*P* = 0.015	χ^2^ = 5.89

*Nonparametric test.

**Evaluation of postoperative residual tumor was performed in 52 of 71 patients with IAC involvement and 84 of 122 patients without IAC involvement.

**Fig 2:**
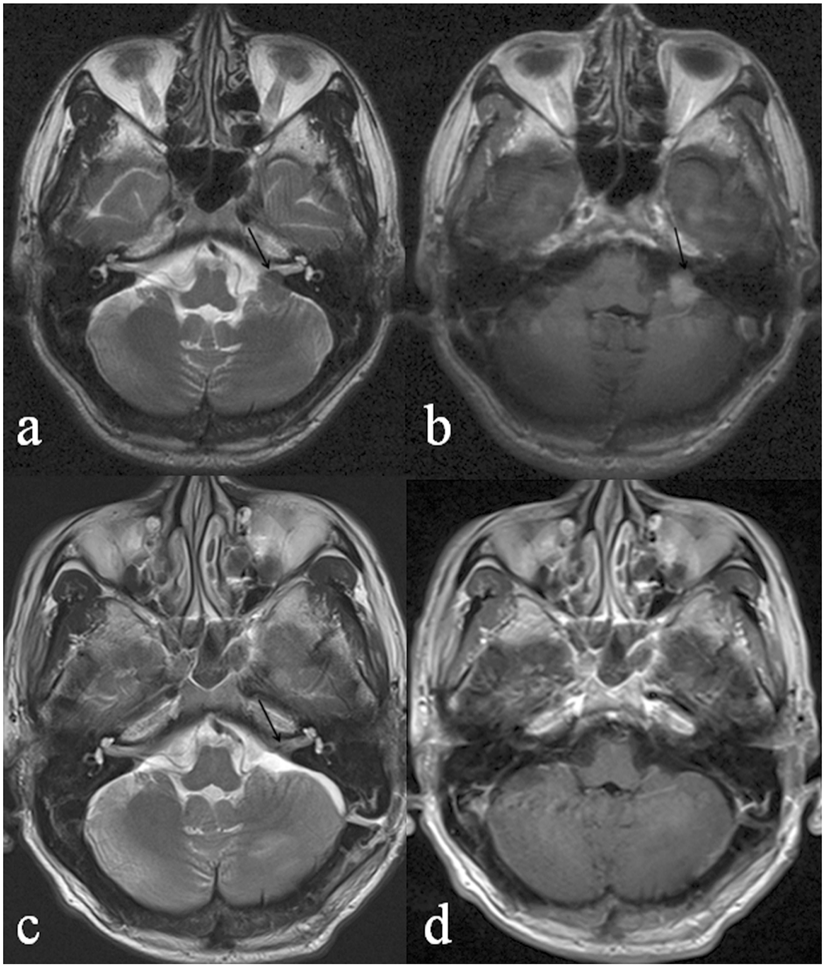
A 60-year-old male patient with mixed meningioma in the left CPA. The preoperative axial T2WI (a) shows an isointense mass in the left CPA. Part of the tumor intrudes into the left IAC (black arrow). The preoperative post-contrast axial image (b) shows an enhanced mass with a maximal axial area of 135mm^2^. The intruding part of the tumor is also enhanced (black arrow). The postoperative axial T2WI (c) shows that the preoperative mass is no longer visible and that the left ICA is essentially uninvolved (black arrow). The postoperative post-contrast axial T1WI (d) shows no enhanced lesion in the CPA and inner opening of the IAC.

**Fig 3:**
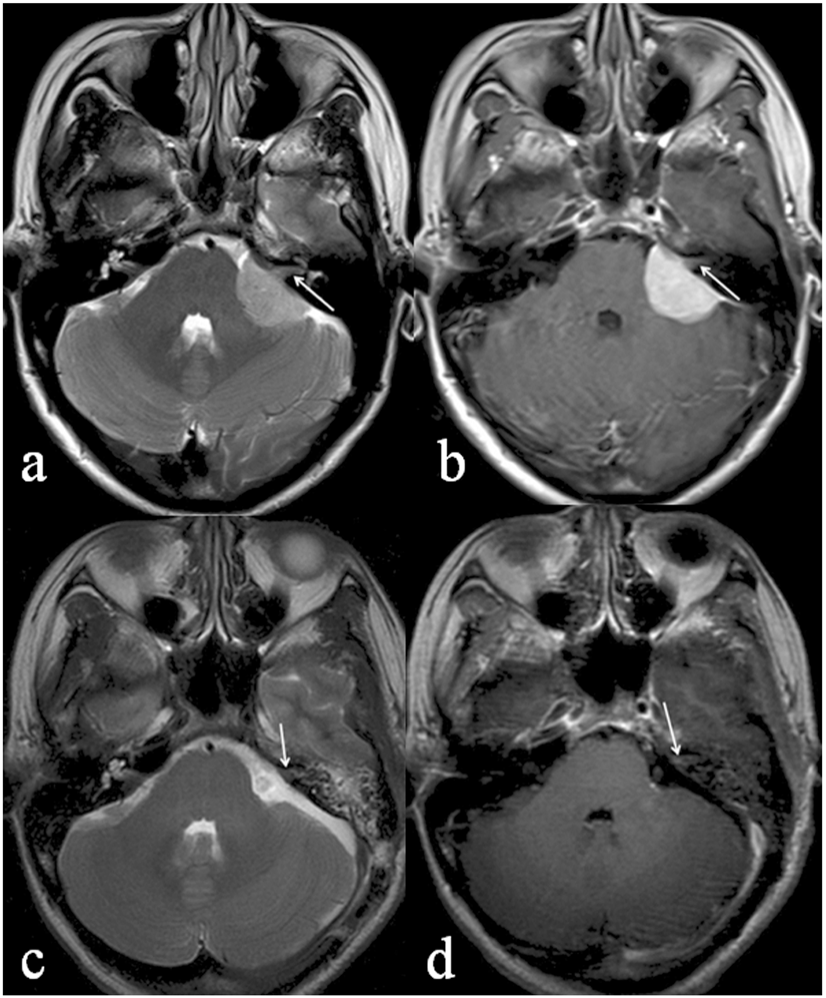
A 43-year-old female patient with mixed meningioma in the left CPA. The preoperative axial T2WI (a) shows the left IAC filled by a hypertintense mass. The left IAC is not dilated and its signal intensity is similar to that of the tumor (white arrow). The preoperative post-contrast axial image (b) demonstrates a heterogeneously enhanced mass with a maximal axial area of 506mm^2^. The left IAC is also slightly enhanced (white arrow). The postoperative axial T2WI (c) shows the absence of the preoperative mass in the left CPA but the IAC is still filled by abnormal signal intensity (white arrow). The postoperative post-contrast axial T1WI (d) reveals heterogeneously enhanced IAC (white arrow).

**Fig 4:**
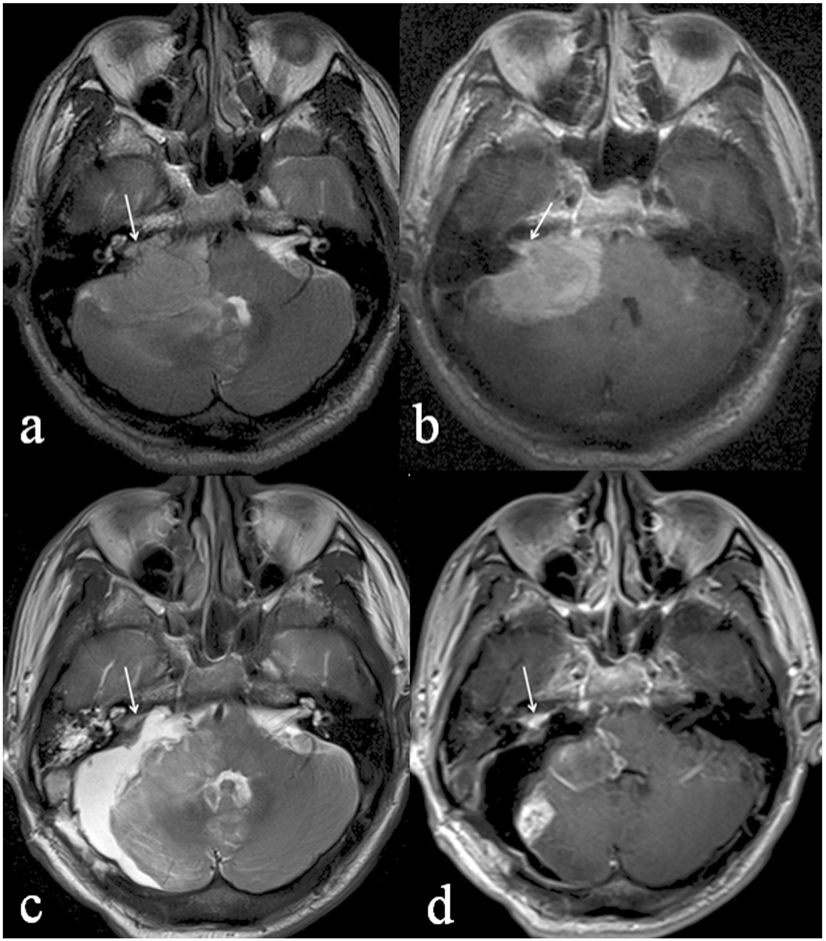
A 55-year-old male patient with meningothelial meningioma in the right CPA. The preoperative axial T2WI (a) shows the right IAC filled and enlarged (white arrow) by a hypertintense mass. Peritumoral edema is seen as patchy high signal intensity in the right cerebellar hemisphere and vermis. The preoperative post-contrast axial image (b) shows a heterogeneously enhanced mass with a maximal axial area of 1104mm^2^. The right IAC is also enhanced (white arrow). The postoperative axial T2WI (c) reveals that the IAC is still filled by abnormal signal intensity (white arrow). The postoperative post-contrast axial T1WI (d) shows heterogeneous enhancement of the enlarged IAC (white arrow).

IAC involvement could be classified into three morphological subtypes, as described in Materials and Methods: intruding (31%, 22/71; Fig 2), filled (29.6%, 21/71; Fig 3), and dilated IAC (39.4%, 28/71; Fig 4). Patients with these tumors differed with respect to age (*P* = 0.021), sex (χ^2^ = 9.404, *P* = 0.009), and postoperative residual tumor (χ^2^ = 7.865, *P* = 0.005) (Table 2). Patients with the intruding form were significantly older than those with either the filled (*P* = 0.01) or the dilated (*P* = 0.02) form. The female: male ratio was significantly higher in patients with filled IAC involvement than in those with intruding involvement (χ^2^ = 9.354, *P* = 0.002). Logistic regression analysis showed that peritumoral edema was a risk factor for IAC involvement (Table 3).

**Table 2:** Characteristics of patients according to IAC involvement subtype.

	Intruding IAC (n = 22)	Filled IAC (n = 21)	Dilated IAC (n = 28)	*P* value	χ2 value
**Age:** in years (mean, range)	54.5± 9.54, 35–69	47.38±7.49, 31–60	47±11.85, 19–66	*P* = 0.021	
**Female patients**: n (%)	12/22 (55)	20/21 (95)	21/28 (75)	*P* = 0.009	χ^2^ = 9.404
**Axial tumor volume in mm** ^**2**^ median (25–75%)	578 (358.75–826)	615 (438.5–818)	759 (400.25–1042.75)	*P* = 0.389^a^	
**Peritumoral edema:** n (%)	9/22(41)	6/21(29)	13/28(46)	*P* = 0.442	χ^2^ = 1.631
**Postoperative residual tumor**: n (%)	7/17(42)	10/11(91)	20/24(83)	*P* = 0.005^b^	χ^2^ = 7.865
**Meningothelial subtype**: n (%)	6/22(27)	13/21(62)	11/28(39)	*P* = 0.066^c^	χ^2^ = 5.448

^a^Nonparametric test.

^b^Patients with intruding vs. filled and dilated IAC involvement.

^c^The meningothelial subtype occurred significantly more frequently in patients with the filled IAC subtype than in patients with intruding IAC (χ^2^ = 1.631, *P* = 0.442).

**Table 3:** Influence of clinical, radiological, and pathological factors on IAC involvement.

	Binary logistic regression		
	B value	Odds Ratio (95%CI)	*P* value
**Age**	0.011	1.011(0.982–1.041)	0.45
**Sex**	0.491	1.633(0.802–3.329)	0.177
**Tumor volume**	0.000	1.000(1.000–1.000)	0.71
**Peritumoral edema**	0.782	2.186(1.157–4.13)	0.016
**Meningothelial subtype**	-0.382	0.682(0.368–1.265)	0.225

## Discussion

Meningiomas are the most common primary intracranial tumor [16, 17]. They usually occur in patients in the fifth decade of life, with a female predominance [11,18,19]. Pathologically, 80–90% of these tumors are benign (WHO Grade I) and the majority are the meningiothelial subtype [20–22]. Consistent with these observations, in the 193 patients in our study, the mean age was 50.2 years and most patients were female (n = 154). The majority (94.8%, 183/193) of the tumors were grade I and the most common pathological subtype was meningothelial (31.6%, 61/193). Meningiomas are generally described based on their anatomic origin (tentorium, superior or inferior petrosal sinuses, petrous ridge dura, IAC, and jugular foramen), but many share a common final location, the CPA, especially meningiomas arising in the posterior fossa[6,23]. According to some authors, CPA meningiomas are mostly meningiomas arising from the petroclival region [13]. In a previous study, CPA meningiomas were classified into three groups, according to the relation of the tumor with respect to the IAC and labyrinth: meningiomas located anterior to the IAC, centered at the IAC, and located posterior to the IAC [24]. Because large meningiomas usually extend into multiple locations, it is often difficult to identify their origin [19]. In our study, CPA meningiomas were regarded as those centered at the IAC, which allowed the establishment of strict and detailed inclusion and exclusion criteria.

In our series, the amount of postoperative residual tumors in patients with the intruding IAC subtype was significantly lower than in patients with the other two subtypes. Thus, in our patients, the greater the IAC involvement, the more difficult it was to achieve total resection of the tumor.

Our study unexpectedly indicated that peritumoral brain edema is a risk factor for IAC involvement by CPA meningioma. Peritumoral brain edema around meiningiomas is vasogenic. While vascular endothelial growth factor is known to play a very important role in edema formation [25], the underlying mechanisms are poorly understood [26, 27] and there is no consensus on the pathological meaning of peritumoral edema. According to some authors, it indicates adhesion of the meningioma to the surrounding parenchyma [11], but to others it does not imply either brain involvement or tumor malignancy [4].

At least in our patients, the three types of IAC involvement had several shared characteristics despite their unique phenotypes. Overall, these tumors demonstrated larger amounts of peritumoral edema, more extensive postoperative residual tumor, and a greater likelihood of being the menigothelial subtype than tumors without IAC involvement. However, patients with IAC intrusion had less residual postoperative tumor than patients with the other two IAC-invasive types, although preoperative tumor volume did not significantly differ among the three subtypes. This may have been partly due to the inclusion in this study only of meningiomas centered around the IAC and our exclusion of tumors spreading from adjacent regions.

Meningiomas are typically classified based on their alteration of the IAC [7, 9], with tumors exhibiting partial IAC intrusion being largely ignored. These latter tumors were included in our study, which is also the first to explore the extent of surgical resection of CPA meningiomas with respect to IAC involvement.

This study has several limitations. First, as a retrospective study, patient selection may have biased the results. Because the cases were identified from a pathological database based on the search terms “meningioma” and “CPA,” the included patients had already undergone a preliminary selection based on their diagnosis of CPA meningioma. This also likely resulted in the exclusion of patients who did not have surgery. Second, the preoperative scanning parameters were not uniform, even though it was confirmed that patients who underwent imaging with the different scanners did not significantly differ in terms of age, sex, and tumor volume, such that scanner type was unlikely to have influenced the subsequent analysis. Third, the tumor volume was represented by its maximal two-dimensional area on the post-contrast axial image, which is only an approximation of the true tumor volume. Fourth, this study did not take into account the effect of the different pathological grades on IAC involvement, because only 10 cases of malignant meningioma in the sample were too small to be analyzed statistically. Fifth, apart from tumor-related morphological changes in the IAC, structural changes in bone, such as hyperostosis, can also occur [6,9]. In our study, the radiological findings were based on MRI, such that changes in bone structure, which are best evaluated using CT, were not analyzed. Finally, we did not consider clinical symptoms and signs or surgical complications in our analysis, although to our knowledge, this was the first study to take into account postoperative residual tumor.

## Conclusions

In summary, this study provides insights into IAC involvement by CPA meningiomas. The results suggest that IAC involvement complicates the surgical resection of these tumors. The presence of peritumoral brain edema increases the risk that meningiomas with IAC involvement will have more extensive postoperative residual tumor.

## References

[1] Ishikawa T, Kawamata T, Kawashima A, Yamaguchi K, Kubo O, Hori T, et al Meningioma of the internal auditory canal with rapidly progressive hearing loss: case report. Neurol Med Chir (Tokyo). 2011;51:233–255. 21441743 10.2176/nmc.51.233

[2] Tomogane Y, Mori K, Izumoto S, Kaba K, Ishikura R, Ando K, et al Usefulness of PRESTO magnetic resonance imaging for the differentiation of schwannoma and meningioma in the cerebellopontine angle. Neurol Med Chir (Tokyo).2013;53:482–489. 23883559 10.2176/nmc.53.482

[3] Alonso Seco A, Polo López R, Labatut Pesce T, Fogué Calvo L. Meningioma of the internal auditory canal: A rare entity. Acta Otorrinolaringol Esp 2010;61:387–388. 10.1016/j.otorri.2009.09.001 19914594

[4] Hamilton BE, Salzman KL, Patel N, Wiggins RH 3rd, Macdonald AJ, Shelton C, et al Imaging and clinical characteristics of temporal bone meningioma. AJNR Am J Neuroradiol. 2006;27:2204–2209. 17110695 PMC7977196

[5] Adachi K, Kawase T, Yoshida K, Yazaki T, Onozuka S. ABC Surgical Risk Scale for skull base meningioma: a new scoring system for predicting the extent of tumor removal and neurological outcome. Clinical article. J Neurosurg. 2009;111:1053–1061. 10.3171/2007.11.17446 19119879

[6] Carney AS, Ward V, Malluci CL, O'donoghue GM, Robertson I, Baldwin DL, et al Meningiomas involving the internal auditory canal: a diagnostic and surgical challenge. Skull Base Surg. 1999;9:87–94. 17171123 10.1055/s-2008-1058154 PMC1656800

[7] Bacciu A, Piazza P, Di Lella F, Sanna M. Intracanalicular meningioma: clinical features, radiologic findings, and surgical management. Otol Neurotol. 2007;28:391–399. 17287658 10.1097/MAO.0b013e31803261b4

[8] von Eckardstein KL, Driscoll CL, Link MJ. Outcome after microsurgery for meningiomas involving the internal auditory canal. Neurosurgery. 2010;67:1236–1242. 10.1227/NEU.0b013e3181efe412 20871449

[9] Asaoka K, Barrs DM, Sampson JH, McElveen JT Jr, Tucci DL, Fukushima T. Intracanalicular meningioma mimicking vestibular schwannoma. AJNR Am J Neuroradiol. 2002;23:1493–1496. 12372737 PMC7976793

[10] Pollock BE, Link MJ, Foote RL, Stafford SL, Brown PD, Schomberg PJ. Radiosurgery as primary management for meningiomas extending into the internal auditory canal. Stereotact Funct Neurosurg. 2004;82:98–103. 15305082 10.1159/000077659

[11] Roser F, Nakamura M, Dormiani M, Matthies C, Vorkapic P, Samii M. Meningiomas of the cerebellopontine angle with extension into the internal auditory canal. J Neurosurg. 2005;102:17–23. 15658091 10.3171/jns.2005.102.1.0017

[12] Kane AJ, Sughrue ME, Rutkowski MJ, Berger MS, McDermott MW, Parsa AT. Clinical and surgical considerations for cerebellopontine angle meningiomas. J Clin Neurosci. 2011;18:755–759. 10.1016/j.jocn.2010.09.023 21507650

[13] Kunii N, Ota T, Kin T, Kamada K, Morita A, Kawahara N, et al Angiographic classification of tumor attachment of meningiomas at the cerebellopontine angle. World Neurosurg. 2011;75:114–121. 10.1016/j.wneu.2010.09.020 21492674

[14] Salehi F, Jalali S, Alkins R, Lee JI, Lwu S, Burrell K, et al Proteins involved in regulating bone invasion in skull base meningiomas. Acta Neurochir (Wien). 2013;155:421–427. 10.1007/s00701-012-1577-9 23238945 PMC3569595

[15] Rousseau A, Mokhtari K, Duyckaerts C. The 2007 WHO classification of tumors of the central nervous system—what has changed? Curr Opin Neurol. 2008;21:720–727. 10.1097/WCO.0b013e328312c3a7 18989119

[16] Fathi AR, Roelcke U. Meningioma. Curr Neurol Neurosci Rep. 2013;13:337 10.1007/s11910-013-0337-4 23463172

[17] Ibebuike K, Ouma J, Gopal R. Meningiomas among intracranial neoplasms in Johannesburg, South Africa: prevalence, clinical observations and review of the literature. Afr Health Sci. 2013;13:118–121. 10.4314/ahs.v13i1.16 23658577 PMC3645104

[18] Raper DM, Starke RM, Henderson F Jr, Ding D, Simon S, Evans AJ, et al Preoperative Embolization of Intracranial Meningiomas: Efficacy, Technical Considerations, and Complications. AJNR Am J Neuroradiol. 2014;35:1798–804. 10.3174/ajnr.A3919 24722303 PMC7966288

[19] Wang DJ, Xie Q, Gong Y, Mao Y, Wang Y, Cheng HX, et al Histopathological classification and location of consecutively operated meningiomas at a single institution in China from 2001 to 2010. Chin Med J (Engl). 2013;126:488–493. 23422112

[20] He S, Pham MH, Pease M, Zada G, Giannotta SL, Wang K, et al A review of epigenetic and gene expression alterations associated with intracranial meningiomas. Neurosurg Focus. 2013; 35:E5 10.3171/2013.10.FOCUS13360 24289130

[21] Kaur G, Sayegh ET, Larson A, Bloch O, Madden M, Sun MZ, et al Adjuvant radiotherapy for atypical and malignant meningiomas: a systematic review. Neuro Oncol. 2014;16:628–636. 10.1093/neuonc/nou025 24696499 PMC3984561

[22] Domingues PH, Sousa P, Otero Á, Gonçalves JM, Ruiz L, de Oliveira C, et al Proposal for a new risk stratification classification for meningioma based on patient age, WHO tumor grade, size, localization, and karyotype. Neuro Oncol. 2014;16:735–747. 10.1093/neuonc/not325 24536048 PMC3984558

[23] Voss NF, Vrionis FD, Heilman CB, Robertson JH. Meningiomas of the cerebellopontine angle. Surg Neurol. 2000;53:439–446. 10874142 10.1016/s0090-3019(00)00195-6

[24] Desgeorges M, Sterkers O, Sterkers JM. Posterior surface of petrous bone meningiomas: Choice of surgical approach and comparison between standard microsurgical techniques and the use of a microscope-guided laser In: Tos M, Thomsen J, eds. Acoustic Neuroma. Amsterdam, the Netherlands: Kugler Publications; 1992.

[25] Hou J, Kshettry VR, Selman WR, Bambakidis NC. Peritumoral brain edema in intracranial meningiomas: the emergence of vascular endothelial growth factor-directed therapy. Neurosurg Focus. 2003;35:E2 10.3171/2013.6.FOCUS13176 24289127

[26] Nassehi D. Intracranial meningiomas, the VEGF-A pathway, and peritumoral brain oedema. Dan Med J. 2013;60:B4626 23651727

[27] Zielinski G1, Grala B, Koziarski A, Kozlowski W. Skull base secretory meningioma. Value of histological and immunohistochemical findings for peritumoral brain edema formation. Neuro EndCurr Neurol Neurosci Rep. 2013;34:111–117. 23645307

